# The Transmembrane Conformation of the Influenza B Virus M2 Protein in Lipid Bilayers

**DOI:** 10.1038/s41598-019-40217-1

**Published:** 2019-03-06

**Authors:** Venkata S. Mandala, Shu-Yu Liao, Martin D. Gelenter, Mei Hong

**Affiliations:** Department of Chemistry, Massachusetts Institute of Technology, 170 Albany Street, Cambridge, MA 02139 USA

## Abstract

Influenza A and B viruses cause seasonal flu epidemics. The M2 protein of influenza B (BM2) is a membrane-embedded tetrameric proton channel that is essential for the viral lifecycle. BM2 is a functional analog of AM2 but shares only 24% sequence identity for the transmembrane (TM) domain. The structure and function of AM2, which is targeted by two antiviral drugs, have been well characterized. In comparison, much less is known about the structure of BM2 and no drug is so far available to inhibit this protein. Here we use solid-state NMR spectroscopy to investigate the conformation of BM2(1–51) in phospholipid bilayers at high pH, which corresponds to the closed state of the channel. Using 2D and 3D correlation NMR experiments, we resolved and assigned the ^13^C and ^15^N chemical shifts of 29 residues of the TM domain, which yielded backbone (φ, ψ) torsion angles. Residues 6–28 form a well-ordered α-helix, whereas residues 1–5 and 29–35 display chemical shifts that are indicative of random coil or β-sheet conformations. The length of the BM2-TM helix resembles that of AM2-TM, despite their markedly different amino acid sequences. In comparison, large ^15^N chemical shift differences are observed between bilayer-bound BM2 and micelle-bound BM2, indicating that the TM helix conformation and the backbone hydrogen bonding in lipid bilayers differ from the micelle-bound conformation. Moreover, H^N^ chemical shifts of micelle-bound BM2 lack the periodic trend expected for coiled coil helices, which disagree with the presence of a coiled coil structure in micelles. These results establish the basis for determining the full three-dimensional structure of the tetrameric BM2 to elucidate its proton-conduction mechanism.

## Introduction

**Figure 1:**
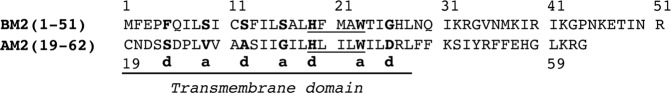
Comparison of the amino acid sequences of the transmembrane region and part of the cytoplasmic region of AM2 and BM2 proteins^24^. Residues at heptad positions *a* and *d*, which represent pore-lining residues, are bolded, and the functionally crucial HxxxW motif is underlined.

Influenza and pneumonia cause 9 to 35 million cases of infection in humans and over 55,000 deaths each year in the US^1^. Flu infection is caused by influenza A and B viruses, with the B strain dominating in the spring months of the flu season. Essential to the lifecycle of both viruses is the integral membrane protein M2, an acid-activated tetrameric proton channel^2–4^. AM2 and BM2 are thus important targets for anti-influenza therapies. The antiviral drugs amantadine and rimantadine inhibit AM2 by blocking its transmembrane (TM) pore^5–8^, but so far BM2 is not inhibited by any drug, owing to the very different amino acid sequence of the BM2 TM domain from that of AM2 (Fig. 1). Mutagenesis and proton-current measurements indicated that the AM2-TM domain has predominantly hydrophobic pore-facing residues^9^ whereas the BM2-TM pore is lined by three polar serines (Ser9, Ser12, and Ser16) (Fig. 1)^10^. The only conserved sequence element between the two TM domains is a HxxxW motif, in which histidine (His) is the proton-selective residue and tryptophan (Trp) is the gating residue^3,11^. Mutation of BM2 His19 abolishes proton conduction^12^ whereas mutation of AM2 His37 disrupts proton selectivity and acid activation^2,13,14^. Despite the conserved HxxxW element, AM2 and BM2 exhibit various differences in their proton conduction profiles. Liposomal proton-flux measurements indicate BM2 conducts protons twice as fast as AM2^15^. Whole-cell electrophysiology data^16^ showed that the two proteins have similar inward proton current at negative voltages, but at positive voltages BM2 conducts protons *outward* whereas AM2 does not^16^. Consistent with these functional differences, solid-state NMR data indicate that His19 in BM2 protonates with significantly lower proton-dissociation equilibrium constants (pK_*a*_’s) compared to His37 in AM2^17^ and to a peripheral His27 in BM2^18^.

Crucial to understanding the proton-conduction and drug-binding differences between AM2 and BM2 are the three-dimensional structures of the proteins in lipid bilayers. It is important to study M2 in lipid bilayers because the small size and oligomeric nature of these proteins make them susceptible to structural perturbations by the membrane environment^19^. The atomic structure of AM2 has been extensively studied^20^, but the structure of BM2 in lipid bilayers is not known. So far the only structure of BM2-TM was solved in DHPC micelles using solution NMR^15^ and showed a coiled coil tetramer that is distinct from the straight four-helix bundle of AM2-TM determined by X-ray crystallography^7,21–23^, solid-state NMR^8,24^ and solution NMR^25^. Even the distinct dimer-of-dimers structure of S31N-AM2, which was solved in DPhPC bilayers, shows straight helices^26^. In principle, the low sequence identity of 24% between BM2 and AM2 TM domains may cause structural differences. However, proteins with low sequence homology but similar functions often adopt similar three-dimensional structures. For example, the G-protein coupled receptors rhodopsin and A_2A_ adenosine receptor^27^ have a low sequence identity of 22% but show backbone RMSDs of 0.27 Å for individual helices. The potassium channels KcsA and MthK have 38% sequence identity for the selectivity filter and inner helix region but adopt the same fold^28,29^.

Site-specific distances measured in bilayer-bound BM2-TM peptides suggest that the BM2-TM structure in detergent micelles may not apply to lipid bilayers. For example, the micelle-bound BM2(1–33) structure shows a pore-facing Phe5^15^ whereas inter-helical distances in lipid bilayers indicate outward-facing phenylene rings^17^. The distance between Trp23 sidechain 5-^19^F and His19 Cα in the HxxxW motif is ~1.7 Å shorter in lipid bilayers than in micelles^30^ whereas the distance between Gly26 C′ and Trp23 5-^19^F is ~1.5 Å longer in lipid bilayers than in micelles. These differences suggest that the micelle-bound BM2-TM structure may not reflect the protein structure in lipid bilayers, due to either micelle perturbation of the protein structure^31^ or uncertainties in the structure determination.

Here we report the determination of the backbone conformation of BM2 in phospholipid bilayers using 2D and 3D correlation solid-state NMR (SSNMR) spectroscopy. We have resolved and assigned the ^13^C and ^15^N chemical shifts of most of the TM residues, using a construct that contains the full TM domain and part of the cytoplasmic domain. The assigned chemical shifts yielded (ϕ, ψ) torsion angles of the TM segment in lipid bilayers at neutral pH. We find that BM2-TM adopts relatively uniform α-helical torsion angles that are very similar to the AM2 TM conformation, suggesting that the proton-conduction differences between the two channels mainly result from the amino acid sidechains. Interestingly, the bilayer-bound BM2-TM has very different ^15^N chemical shifts as well as different helical length from micelle-bound BM2, indicating that the membrane environment exerts a significant influence on the conformation of this proton channel.

## Results and Discussion

### Biophysical characterization and membrane-dependent conformation of BM2(1–51)

We chose BM2(1–51) (Fig. 1) as the construct for conformational analysis because this domain includes the TM domain required for proton conduction as well as the necessary cytoplasmic segment for membrane targeting of the protein. In virus-infected MDCK cells, a BM2 deletion mutant that lacks residues 24–50 remained in the Golgi bodies, whereas a deletion mutant lacking residues 51–80 migrated to the plasma membrane like full-length BM2^4^. The construct excludes the BM1-interacting domain, which resides between residues 84–108, as shown by chemical shift perturbations^15^. We were not able to cleave the N-terminal solubility tag used for protein expression; however, the tag is disordered and highly mobile, as it exhibits narrow signals in the ^13^C INEPT spectrum (*vide infra*), thus it does not interact with the TM helix. To simplify the NMR spectra, Ile residues are not labeled.

**Figure 2:**
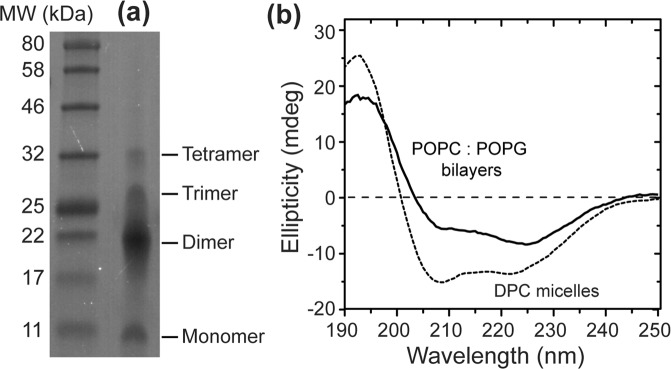
Purification and biophysical characterization of TEV-BM2(1–51). (**a**) SDS-PAGE gel of purified BM2, which runs as a mixture of monomers to tetramers under denaturing conditions. The molecular weight of the whole construct is 9.8 kDa. (**b**) CD spectra of BM2 in 0.5% DPC micelles and POPC: POPG bilayers at pH 7.5. Both spectra exhibit two minima at 209 nm and 222 nm that are characteristic of the α-helical conformation.

SDS-PAGE gel analysis (Fig. 2a, Supplementary Fig. S1) shows that purified BM2(1–51) runs as a mixture of monomer, dimer, trimer and tetramer under denaturing conditions. This is consistent with fluorescence resonance energy transfer data and chemical crosslinking data that indicate that BM2 is a tetrameric membrane protein^32,33^. Thus, the solubility tag does not perturb the oligomeric assembly of BM2(1–51). Circular dichroism (CD) spectra of BM2(1–51) in DPC micelles and in POPC:POPG (4:1) bilayers (Fig. 2b) show two minima at 209 nm and 222 nm, characteristic of the α-helical conformation^34^. Spectral deconvolution yielded ~30% α-helix, ~15% β-sheet, ~15% turn, and ~40% disordered structure for DPC-bound protein, whereas in lipid bilayers, the fractions are ~20% α-helix, ~30% β-sheet, ~15% turn, and ~35% disordered structure. The 35–40% disordered structure is consistent with the fact that the solubility tag represents ~40% of the whole construct, and that the helical conformation dominates in the native BM2 sequence. Indeed, CD spectra of BM2(1–51) without a solubility tag, produced by native chemical ligation, have recently been reported and indicate a helical content of ~60% in POPC vesicles^35^. These comparisons indicate that the TM conformation of BM2 is unaffected by the solubility tag.

**Figure 3:**
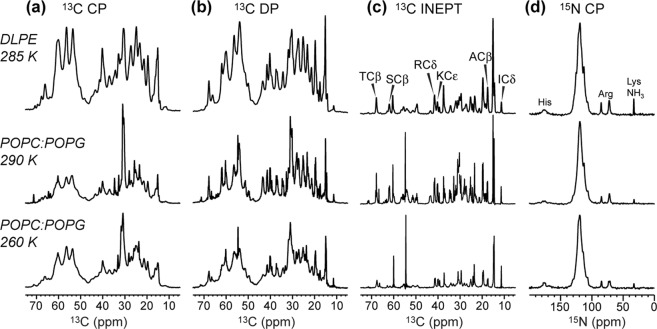
1D ^13^C and ^15^N NMR spectra of BM2 in DLPE and POPC: POPG (4:1) bilayers at different temperatures. (**a**) ^13^C CP spectra, which selectively detect immobilized residues. (**b**) ^13^C DP spectra, which detect all residues’ signals. (**c**) ^13^C INEPT spectra, which selectively detect the signals of highly dynamic residues. (**d**) ^15^N CP spectra of immobilized residues. Top row shows the DLPE spectra measured at 285 K. Middle row shows the POPC: POPG spectra measured at 290 K. Bottom row shows the 260 K spectra of the POPC: POPG sample. Signals from the N-terminal solubility tag are predominantly seen in the INEPT spectra, whereas the TM signals dominate the CP spectra.

To investigate residue-specific conformation of BM2(1–51) in phospholipid bilayers, we first measured 1D ^13^C and ^15^N spectra of the protein in DLPE and POPC:POPG (4:1) membranes (Fig. 3). DLPE has been shown to support a well ordered conformation of AM2^36^ due to the hydrogen-bonding ability of the phosphatidylethanolamine (PE) headgroup and the saturated nature of the two acyl chains (12:0). As a result, DLPE has a high phase-transition temperature of 29 °C compared to the POPC/POPG transition temperature of −2 °C^37^. Indeed, DLPE-bound BM2(1–51) shows strong protein signals in the ^13^C CP spectrum at 285 K, whereas the POPC/POPG sample shows weak protein signals at a similar temperature. Decreasing the temperature to 260 K increased the sensitivity of the POPC/POPG sample but also broadened the ^13^C linewidths. Therefore, the DLPE membrane gives higher sensitivity as well as resolution for the protein spectra. The ^13^C DP spectra (Fig. 3b), which detect both dynamic and rigid residues, show narrow ^13^C signals superimposed with broader peaks. These narrow signals are preferentially detected in the ^13^C INEPT spectra (Fig. 3c) and arise from the solubility tag and residues 34–51 in the native BM2, based on amino-acid type assignment and the absence of these signals in dipolar-based spectra. For example, we detected Ala Cβ, which is present in the solubility tag but not BM2(34–51), and Ile Cδ, which is present in BM2(34–51) but not the solubility tag. We also observed Ser Cβ, Thr Cβ, Arg Cδ, and Lys Cε, which are present in both the tag and BM2(34–51). Finally, ^15^N CP spectra of the two bilayer samples show similar spectral resolution (Fig. 3d): in addition to the main amide ^15^N peak, a His sidechain peak at ~170 ppm, Arg guanidinium ^15^N signals at ~85 ppm and ~72 ppm, and a Lys NH_3_ peak at ~33 ppm are observed.

Taken together, these 1D spectra indicate that DLPE and POPC/POPG bilayers produce similar conformation of BM2 but different dynamics: the DLPE-bound protein is more immobilized than the POPC/POPG-bound protein due to the higher phase-transition temperature of DLPE bilayers. This is confirmed by 2D ^13^C-^13^C correlation spectra of the two samples (Supplementary Fig. S2), which show similar chemical shifts for most resonances, but with additional cross peaks in the DLPE spectrum, which can be assigned to Lys32, Arg33, Val35 and inter-residue correlations in the TM domain. Given the similarity of the chemical shifts in the two membranes, we focused on the DLPE sample for resonance assignment.

### ^13^C and ^15^N chemical shift assignment of bilayer-bound BM2

**Figure 4:**
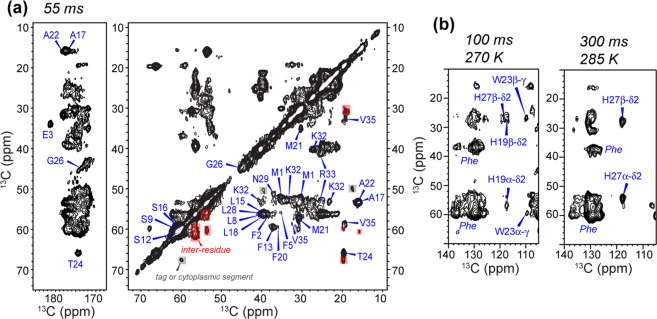
2D ^13^C-^13^C correlation spectra of DLPE-bound BM2. (**a**) Aliphatic and carbonyl regions of the 2D spectrum measured with 55 ms ^13^C spin diffusion. The ^13^C linewidth is 0.8–1.1 ppm. (**b**) Aliphatic-aromatic regions of the 2D spectra measured with 100 ms (*left*) and 300 ms (*right*) ^13^C spin diffusion. The functionally important H19 and W23 in the HxxxW motif and the C-terminal H27 are assigned.

**Figure 5:**
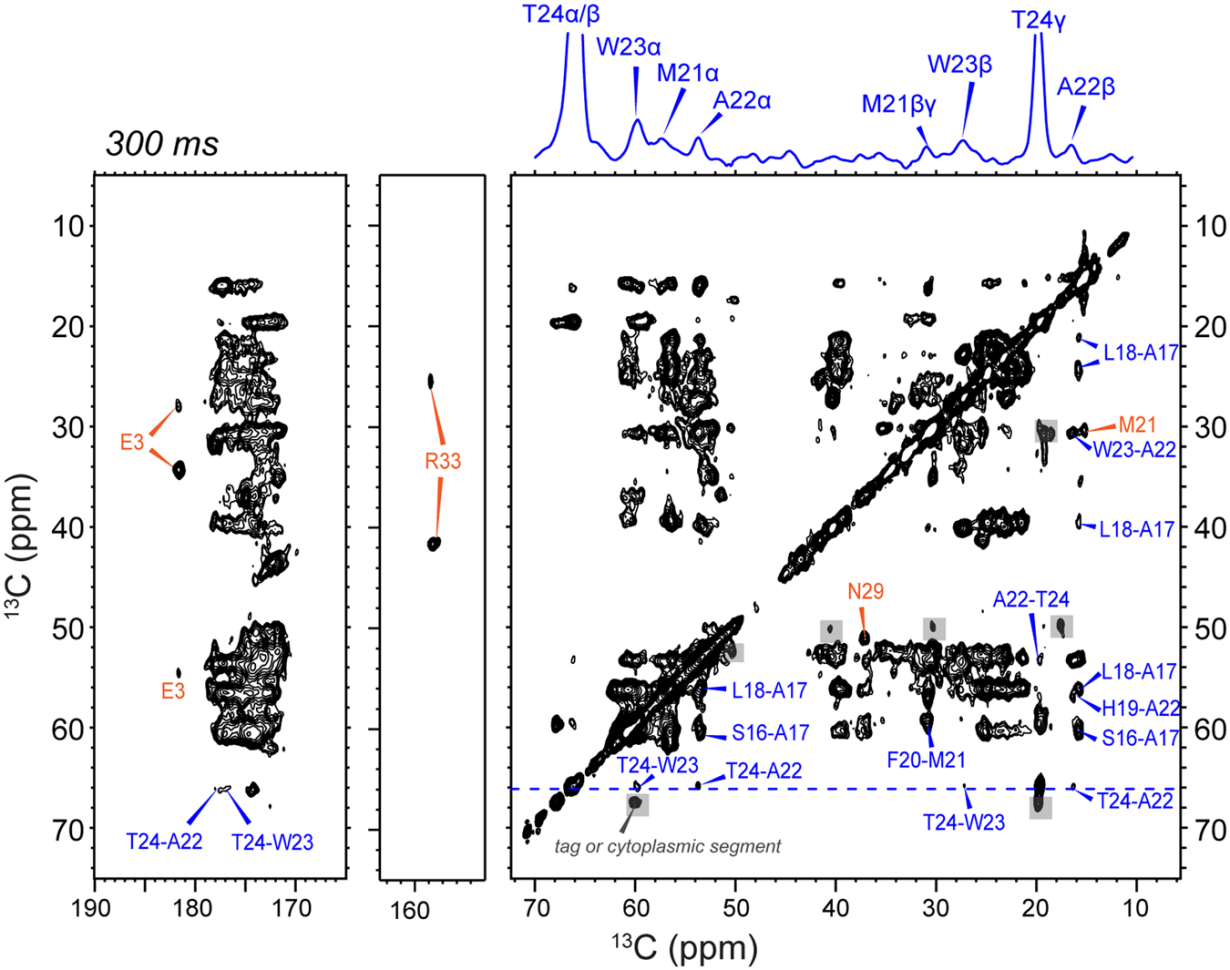
Aliphatic and carbonyl regions of the 300 ms 2D ^13^C-^13^C correlation spectrum, showing inter-residue cross peaks (*blue*) and long-range intra-residue cross peaks (*orange*). Resolved peaks such as E3 Cδ were used to assign the other ^13^C signals of the same residue. Residues such as A17, A22 and T24 show cross peaks to multiple neighboring residues, which validate sequential assignments of the 3D NCACX and NCOCX spectra. The ^13^C slice at the T24 Cα and Cβ position is shown above the 2D spectrum to illustrate cross peaks to neighboring residues.

Figure 4a shows the 2D ^13^C-^13^C correlation spectrum of DLPE-bound BM2(1–51) measured with a ^13^C spin diffusion mixing time of 55 ms. Most cross peaks in this dipolar-based spectrum show α-helical chemical shifts, except for a few resonances that can be assigned to the extra-membrane region. The ^13^C linewidths are 0.8–1.1 ppm, which are similar to the linewidths of AM2-TM in the absence of amantadine^38,39^. Only a single set of cross peaks is observed, indicating a single conformation. This differs from AM2-TM, which shows multiple peaks for specific residues such as Ser31 and Gly34, indicating local conformational polymorphism^38,39^. With longer mixing times numerous additional cross peaks are observed (Fig. 4b), which allowed sequential assignment of many residues even without 3D ^15^N-^13^C correlation experiments. For example, with 100 ms and 300 ms mixing, aromatic-aliphatic cross peaks are observed for His19, Trp23 and His27 (Fig. 4b). At 300 ms, the aliphatic region of the 2D spectrum (Fig. 5) shows well-resolved sequential cross peaks. For example, the characteristic Thr24 Cα and Cβ peaks show correlations with Trp23, Ala22 and Met21, which are assigned based on their aromatic and Cβ chemical shifts. The well-resolved Ala17 and Ala22 Cβ chemical shifts allowed the assignment of their neighboring residues such as the Ser16-Ala17-Leu18 triplet and the Met21-Ala22-Trp23-Thr24 quartet. The parallel homo-tetrameric assembly of M2 limits intramolecular and intermolecular cross peaks to neighboring residues in the protein sequence. Finally, the resolved chemical shifts of Glu3 Cδ in the carbonyl region and Arg33 Cζ at 157.5 ppm allowed the assignment of these spin systems as well as their neighboring residues such as Pro4 and Phe2.

**Figure 6:**
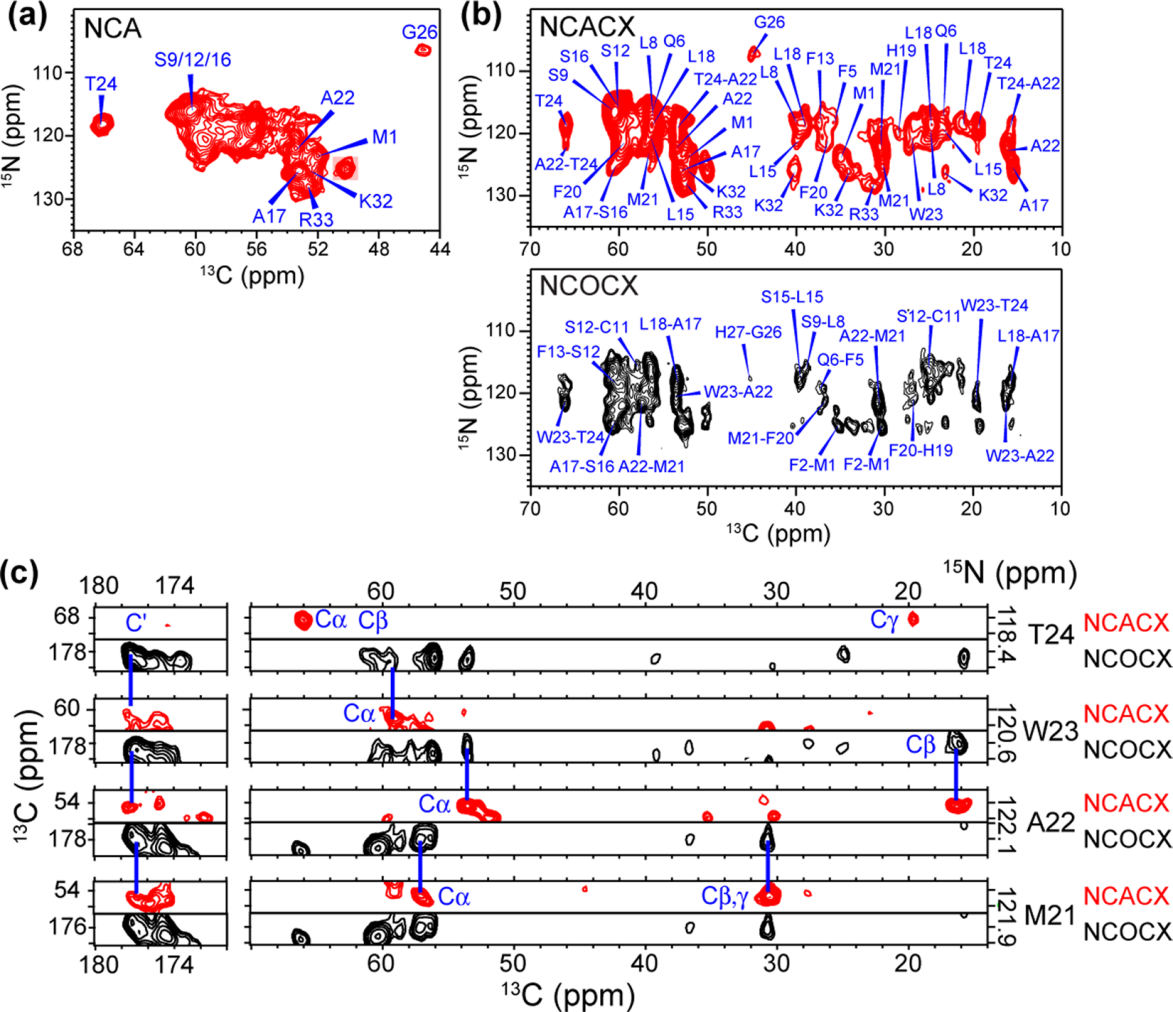
2D and 3D ^15^N-^13^C correlation spectra of DLPE-bound BM2. (**a**) 2D NCA correlation spectrum measured using 4 ms ^SPECIFIC^CP mixing. The ^15^N linewidths are 2.1–2.6 ppm. (**b**) 2D ^15^N-(^13^CA)-^13^CX spectrum measured using 1.4 ms TEDOR mixing and 110 ms ^13^C-^13^C CORD mixing, and 2D ^15^N-(^13^CO)-^13^CX spectrum measured using 4 ms ^SPECIFIC^CP contact and 150 ms ^13^C-^13^C DARR mixing. Complete resonance assignment is shown for the N(CΑ)CX. Several peaks overlapped in the 2D spectrum are resolved in the 3D NCACX spectrum. (**c**) Representative strips from the 3D NCACX (*red*) and NCOCX (*black*) spectra, showing sequential assignment of residues T24-F20.

To complete the sequential assignment of all labeled residues in BM2(1–51), we measured 2D and 3D ^15^N-^13^C correlation spectra. The 2D NCA, N(CΑ)CX and N(CO)CX correlation spectra show ^15^N linewidths of 2.1–2.6 ppm (Fig. 6a,b), which are similar to the ^15^N linewidths of AM2-TM without the drug^38,40,41^. 3D NCACX and NCOCX spectra removed the remaining spectral overlap and allowed the majority of the ^13^C and ^15^N signals of the TM residues to be resolved and assigned. A representative 3D strip of peak connectivities from Thr24 to Met21 is shown in Fig. 6c. The three serine residues have overlapping Cα and Cβ chemical shifts but different N and C′ chemical shifts, which allowed them to be assigned by their connectivities to neighboring residues such as Ala17 and Cys11. In total we assigned 29 residues out of 43 labeled residues in the protein (Supplementary Table S1). The unassigned residues are clustered to the segment Asn36 to Arg51, indicating that they are too mobile to be detected in the CP-based 2D and 3D spectra.

### Backbone conformation of BM2-TM in lipid bilayers versus detergent micelles

The assigned ^13^C and ^15^N chemical shifts are a direct reporter of the backbone (φ, ψ) torsion angles. We computed the secondary chemical shifts of Cα, Cβ, and C′ and N of all assigned residues using the TALOS-N software to define the TM α-helix boundary. Positive secondary shifts for Cα and C′ and negative shifts for Cβ and N indicate α-helical conformation whereas negative Cα and C′ secondary shifts and positive Cβ and N secondary shifts indicate β-sheet conformation^42,43^. Values near zero indicate random coils. Note that Ile residues are not labeled in the protein.

**Figure 7:**
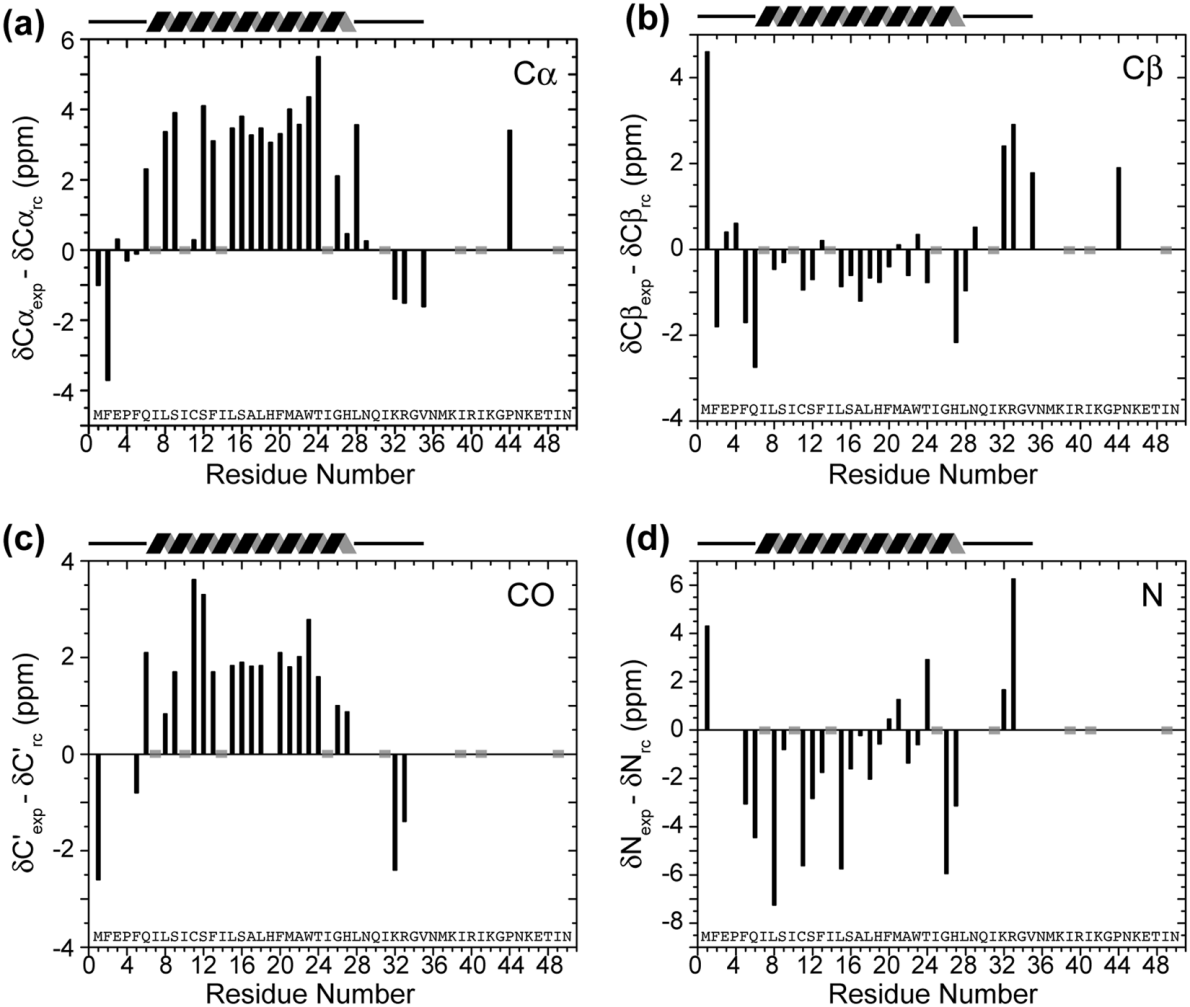
Secondary shifts of DLPE-bilayer bound BM2 for (**a**) Cα, (**b**) Cβ, (**c**) C′ and (**d**) N, calculated using TALOS-N. Positive values for Cα and C′ and negative values for Cβ and N indicate α-helical conformation, whereas the opposite indicates β-sheet conformation. Grey shaded bars at zero secondary shifts indicate the positions of unlabeled Ile residues. The secondary shifts indicate an α-helix between residues 6 and 28. Residues 1–5 and 29–35 are disordered whereas residues 35–51 are not visible in dipolar-based spectra, indicating they are dynamic.

**Figure 8:**
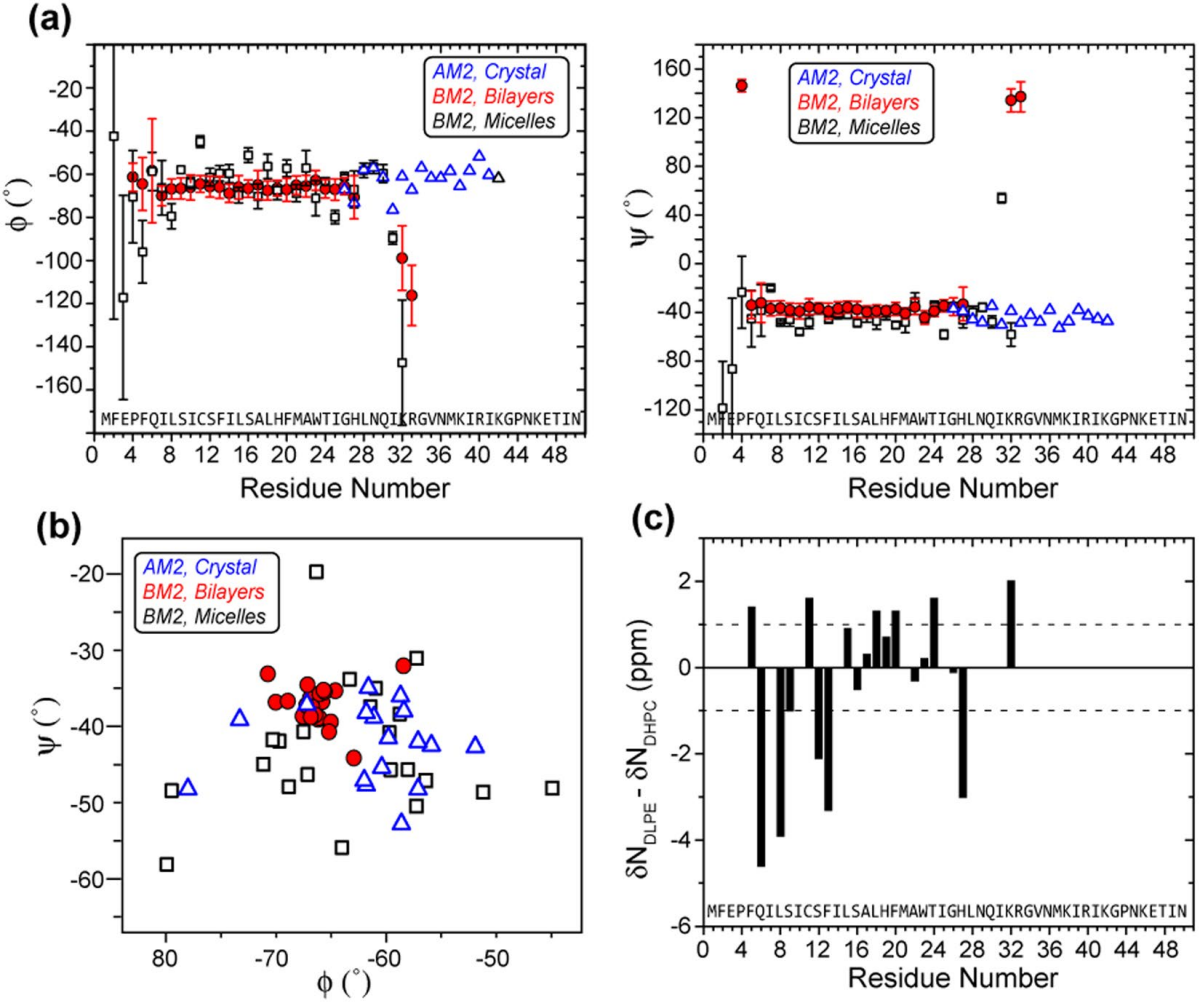
(ϕ, ψ) Torsion angles of BM2. (**a**) TALOS-N predicted (φ, ψ) angles (*red*) of BM2 in lipid bilayers, where error bars indicate the precision of the prediction. These are overlaid with the torsion angles of the solution structure of DHPC-bound BM2 (*black*)^15^ and with the torsion angles of the crystal structure of AM2-TM in lipid cubic phase (*blue*)^22^. All three structures were measured at high pH, corresponding to the closed state of the channel. (**b**) Ramachandran diagram of BM2-TM and AM2-TM. (**c**) ^15^N chemical shift differences between bilayer-bound BM2 and micelle-bound BM2. About half of the residues show chemical shift differences of >1 ppm.

Figure 7 shows strongly positive secondary shifts for Cα and C′ and negative secondary shifts for Cβ and N for residues 6–28, thus defining the core of the TM helix. Except for Cys11 and His27, which have small Cα secondary shifts of 0.3 and 0.5 ppm, all other Cα secondary shifts are larger than 2 ppm, with the largest secondary shift observed for Thr24 (5.5 ppm). In comparison, segments Met1-Phe5 and Asn29-Val35 show random coil or β-sheet secondary shifts in the DLPE bilayer. Therefore, the helical core of BM2-TM in lipid bilayers spans residues 6–28, while the other residues are either disordered or adopt minor β-sheet conformations. Although DLPE bilayers are ~4 Å thinner than POPC bilayers^44,45^, the membrane thickness generally has little effect on the helix length but mainly influences the helix orientation through hydrophobic matching^46–48^, thus we expect the helical conformation to be unchanged in POPC: POPG bilayers. This BM2 TM helix length is in good agreement with cysteine scanning mutagenesis data of full-length BM2 in oocytes^10^, which indicated an α-helix from residues L8 to H27 with a repeat of 3.4 ± 0.1 residues per turn. In comparison, the BM2-TM helix length in DHPC micelles is noticeably longer, extending from Glu3 to Ile31^15^. Moreover, the ^15^N chemical shifts of micelle-bound BM2^15^ differ significantly from the ^15^N chemical shifts of bilayer-bound BM2: half of the TM residues (e.g. S9, S12, F20 and T24) exhibit ^15^N chemical shift differences larger than 1 ppm and residues near the two ends of the TM domain (e.g. Q6 and H27) have even larger ^15^N shift differences of 3.0–4.6 ppm (Fig. 8c, Supplementary Table S2). The root-mean-square deviation of the ^15^N chemical shifts is 2.05 ppm. These large ^15^N chemical shift differences could be due to differences in backbone conformation, amide hydrogen bonding, or a combination thereof. The increased ^15^N chemical shift differences at the ends of the TM domain, coupled with the random coil or β-sheet ^13^C chemical shifts of residues 1–5 and 32–33, indicate a significantly different backbone conformation for these residues compared to the extended α-helix seen in micelle-bound BM2. The ^13^C chemical shifts of micelle-bound BM2 are not available in databases to allow further comparison with the bilayer values^15^; but the significant ^15^N chemical shift differences already indicate that BM2-TM has a different backbone conformation and perhaps backbone hydrogen bonding in lipid bilayers than in micelles.

**Figure 9:**
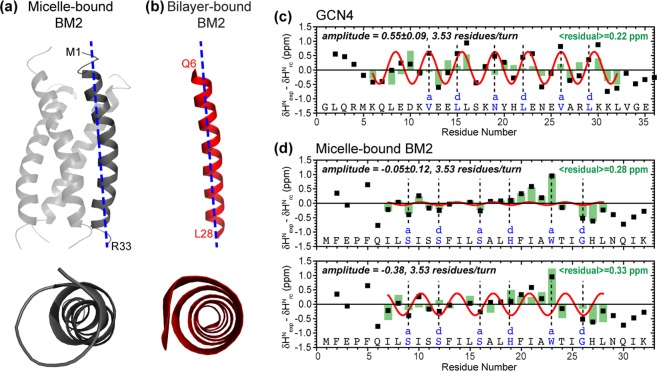
Comparison of the backbone conformations of the BM2 TM helix in (**a**) DHPC micelles (PDB code: 2KIX, *grey*) and (**b**) DLPE lipid bilayers (*red*). The latter is a conformational model using chemical-shift derived (ϕ, ψ) torsion angles. (**c**) H^N^ secondary chemical shifts of GCN4 coiled coil. A periodicity of 3.5 residue/turn is observed. Residues at inward-facing *a* and *d* positions (dashed lines) show positive secondary shifts of about +0.5 ppm whereas those at outward-facing positions show negative secondary shifts of about −0.5 ppm. Best-fit oscillations are shown in red and the differences between fit and experimental values are shown in green. (**d**) H^N^ secondary chemical shifts of micelle-bound BM2. No periodicity of H^N^ secondary shift is present, and pore-facing Ser9, Ser12, and Ser16 (dashed lines) show negative secondary shifts, both contradicting a coiled coil structure. A best fit using 3.53 residues/turn gave a small amplitude and an RMSD of 0.28 ppm between the fit and the experimental chemical shifts. When a larger amplitude is used for the sinusoidal fit, the RMSD does not improve. Therefore, the reported coiled coil structure for micelle-bound BM2 is inconsistent with the H^N^ chemical shifts.

We used TALOS-N to predict the (ϕ, ψ) torsion angles of residues 4–27 and 32–33 from the measured chemical shifts (Supplementary Table S3, Fig. 8a**)**. The φ angles range from −58.4° to −70.7°, with an average of −65.9 and a standard deviation of 2.5° for the distribution. The ψ angles range from −32.0° to −44.1°, with an average of −37.0 and a similarly small standard deviation of 2.6° for the distribution. These (ϕ, ψ) angles are in good agreement with the ideal α-helical torsion angles of (−65°, −40°). The precisions of the TALOS-N predictions, defined as one standard deviation for the (φ, ψ) angles among the best-matched peptides for each residue, are shown as error bars in Fig. 8a, and average 6.1° for φ and 6.5° for ψ for all residues. The accuracy of the predictions, defined as the RMSD from the crystallographic torsion angles, is ~12°. Although the uncertainty of TALOS-N precludes a high-resolution definition of the helix conformation without additional distance restraints, these chemical-shift derived (ϕ, ψ) angles in bilayers are in good agreement with the latest 1.1 Å crystal structure of AM2-TM^22^, whose (ϕ, ψ) angles are (−62.3 ± 6.1°, −43.8 ± 5.3°). In comparison, the reported BM2-TM structure in DHPC micelles has significantly larger (ϕ, ψ) variations of 8–11°, with values of (−64.8 ± 10.6°, −43.2 ± 8.3°) (Fig. 8b). The (ϕ, ψ) angles of BM2-TM domain in micelles and bilayers are shown in Fig. 9(a,b), where the reported micelle-bound structure has a coiled coil shape.

Large torsion-angle variations in a helix can result from a coiled coil or a generally distorted helix. Many high-resolution coiled coil structures are known. For example, the 1.8 Å-resolution structure of the trimeric GCN4 leucine zipper exhibits backbone torsion angles of (ϕ = −64.5 ± 7.2°, ψ = −39.9 ± 8.8°)^49–51^. The fusion peptide of gp41 (residues 282–304)^52^ in DHPC micelles, constrained by residual N-H dipolar couplings, exhibits torsion angles of (ϕ = −55 ± 11°, ψ = −43 ± 12°). The coiled coil domain of the mitochondrial division protein 1 has torsion angles of (ϕ = −64.1 ± 12.5°, ψ = −40.0 ± 13.9°)^53^. These significant torsion angle variations (~10°) reflect the systematic shortening of main-chain hydrogen bonds on the inward-facing (‘concave’) side of the coiled coil compared to the outward-facing (‘convex’) side.

The most direct NMR structural parameter to distinguish coiled coils from straight helices are residual dipolar couplings, because helix curvature is directly reflected by N-H bond orientations. This approach was demonstrated for the gp41 fusion peptide^52^, which forms a curved helix in DHPC micelles but a straight helix in lipid bicelles. Compared to residual dipolar couplings, intramolecular distance restraints are relatively insensitive to helix curvature, whereas intermolecular distance restraints can capture helix curvature, since the two ends of a helix should be adjacent to different faces of the second helix. Compared to these orientational restraints and intermolecular distance restraints, ^13^C and ^15^N chemical shifts in general cannot distinguish a straight helix from a coiled coil. In contrast, amide proton (H^N^) chemical shifts are a direct reporter of coiled coils: the concave side of the curved helix have downfield shifted H^N^ chemical shifts compared to the more weakly hydrogen-bonded amides on the convex side^54–56^. This trend is exemplified by the canonical GCN4, which shows periodic H^N^ secondary chemical shifts^57,58^ with a periodicity of 3.53 residues/turn (Fig. 9c). Residues at inward-facing *a* and *d* positions of the heptad repeat have positive H^N^ secondary shifts of about +0.5 ppm whereas residues at outward-facing *c* and *f* positions show negative secondary shifts of about −0.5 ppm. A secondary shift of 0.5 ppm corresponds to a difference in hydrogen-bond length (R_N…O_) of ~0.2 Å^59^, which is consistent with the variation in R_N…O_ in the GCN4 crystal structure^51^. These periodic H^N^ secondary shifts are also seen in other coiled coil proteins such as the antiparallel bovine IF_1_^60^ and the parallel myosin-binding subunit^61^.

In summary, a coiled coil helix should manifest periodic H^N^ secondary chemical shifts, and accurate determination of coiled coil structures by NMR requires orientational restraints, followed by intermolecular distance restraints. However, the micelle-bound BM2-TM does not show any periodic H^N^ secondary shifts^15^ (Fig. 9d), and the pore-facing Ser9, Ser12, and Ser16 have *negative* H^N^ secondary shifts, both contradicting the coiled coil structure. The structure was also not restrained by any residual dipolar couplings but only by NOEs; moreover the 11 NOEs that were attributed to be intermolecular did not result from mixed labeled samples but by their exclusion from intramolecular contacts.

Given that the amide proton secondary shifts of micelle-bound BM2 are inconsistent with a coiled coil, the non-ideality of the BM2-TM helix in micelles, if true, would likely be caused by the micelle environment. The BM2-TM domain may curve itself to fit within the 35–38 Å diameter of the DHPC micelle^62,63^, similar to a number of α-helical membrane proteins whose structures have been solved and compared between micelles and better bilayer mimics. For example, the HIV-1 gp41 fusion peptide (residues 282–304) is coiled in DHPC micelles but straight in DMPC: DHPC bicelles^52^. The TM helices of diacylglycerol kinase (DgkA) have an outward curvature in DPC micelles^64^ but are straight in phospholipid bilayers^31,65^ and lipid cubic phases^66^. DgkA is also 600 times less active in DPC micelles than in LMPC micelles^67^, implicating that structural distortion by micelles adversely impacts function. The TM helix of the *Mycobacterium tuberculosis* protein Rv1761c shows a ~120° kink in DPC micelles^68^, which is absent in lipid bilayers^31^. These examples suggest that micelles have a significant propensity to distort membrane protein structures from those in lipid bilayers.

Determination of the three-dimensional structure of the tetrameric BM2-TM assembly in lipid bilayers to high resolution requires intramolecular and intermolecular distance restraints and orientational restraints. Experiments along these lines are currently in progress. An accurate backbone conformation of BM2-TM in lipid bilayers will impact our understanding of the mechanism of proton conduction, since the positions of sidechains relative to the channel pore must be known correctly. For example, Phe5, which lies at the periphery of the α-helical core of bilayer-bound BM2, has a nearest-neighbor inter-helical distance of 11 Å between the para-hydrogen atoms in lipid bilayers^18^, which is much longer than the ~5 Å distance reported in the micelle-bound BM2 structure. This large opening indicates an outward-facing phenylene ring conformation, which rules out pore obstruction as the cause for the lack of drug binding to BM2, but points to the polar nature of the BM2 pore as the cause. Similarly, ^13^C-^19^F distance measurements in lipid bilayers found that the Gly26 C′ distance to 5-^19^F-Trp23 is much longer than the 6.8 Å distance reported in the solution structure^30^. A straight helix in lipid bilayers would increase the distance ~8.0 Å for the same t-105° rotamer of Trp23, which would be consistent with the REDOR data.

## Conclusions

These SSNMR data indicate that the BM2-TM domain adopts a well-ordered α-helical conformation in lipid bilayers. Residues 6–28 form the helical core, whereas residues 1–5 and 29–35 are unstructured. Based on the assigned ^13^C and ^15^N chemical shifts, the average (φ, ψ) torsion angles of the TM residues are (−65.9°, −37.0°). This backbone conformation is similar to the conformation of AM2-TM, despite only 24% sequence identity. BM2 exhibits a single set of chemical shifts and hence a single TM conformation at high pH. This differs from AM2-TM, which shows local conformational polymorphism at several residues. Large ^15^N chemical shift differences indicate that the α-helical conformation of BM2 in lipid bilayers differs from the micelle-bound conformation. Moreover, the H^N^ secondary shifts of micelle-bound BM2 lack the periodic trend expected for coiled coils, thus disputing the coiled-coil structure in micelles. The bilayer-bound BM2-TM conformation, combined with previously measured distance restraints, indicates that the lack of amantadine binding to BM2 is not due to different three-dimensional structures of the tetrameric channel but is most likely due to the polar nature of the channel pore. Future determination of the complete three-dimensional structure of BM2-TM in lipid bilayers will further elucidate the mechanistic underpinnings for the functional differences between AM2 and BM2 channels, as well as facilitating drug design to inhibit the influenza B virus.

## Methods

### Construction of the recombinant BM2(1–51) protein

The plasmid encoding BM2(1–51) of Influenza B/Maryland/ 1/2001 (MFEPFQIL-SICSFILSALHFMAWTIGHLNQIKRGVNMKIRIKGPNKETINR) was obtained from Integrated DNA Technologies. The gene includes a His_6_ segment and a TEV protease cleavage site (HHHHHHSSGVDLGTENLYFQSNA) before the native BM2(1–51) sequence. The gene was PCR amplified and cloned into a pET21a plasmid^40^ between an N-terminal BamHI restriction site that follow a T7 epitope and a C-terminal EcoRI restriction site. The expression construct thus contains 88 residues, including 37 residues from the T7-His_6_-TEV tag and 51 residues for BM2. The plasmid was transformed into *E*. *coli* BL21 (DE3) cells for expression, and the DNA sequence of the construct was verified by Sanger sequencing.

### Expression and purification of ^13^C, ^15^N-labeled BM2(1–51)

The BM2 construct was uniformly ^13^C, ^15^N labeled for all residues except for Ile. Thus, 43 out of the 51 native BM2 residues are ^13^C and ^15^N-labeled. A glycerol cell swab stored at −70 °C was used to streak an LB-Amp plate and colonies were allowed to grow overnight at 37 °C. A single colony was selected to start a 10 mL LB culture containing 100 μg/mL ampicillin the next night. This starter culture was used to inoculate 1 L of LB media containing 100 μg/mL ampicillin. Cells were grown at 37 °C until an OD_600_ of 0.6–0.8 and harvested by centrifugation for 10 minutes at 20 °C and 7,000 rpm. These LB cells were resuspended in 0.5 L of M9 minimal media (pH 7.8, 48 mM Na_2_HPO_4_, 22 mM KH_2_PO_4_, 8.6 mM NaCl, 4 mM MgSO_4_, 0.2 mM CaCl_2_, 1 mg ampicillin) containing 1 g/L ^15^N-NH_4_Cl, 2 g/L U-^13^C glucose, and 100 mg/L unlabeled Ile for isotopic labeling of BM2. The cells were incubated in M9 media for 30 minutes at 37 °C, then protein expression was induced by addition of 0.4 mM isopropyl β-D-1-thiogalactopyranoside (IPTG). Protein expression proceeded for 5–6 hours, reaching a typical OD_600_ of ~1.1, then cells were spun down at 4 °C and 5000 rpm for 10 min, resuspended in 20 mL lysis buffer (pH 8.0, 50 mM Tris-HCl, 50 mM NaCl), and stored overnight at −70 °C.

For BM2 purification, cells were thawed at 37 °C and lysed at 4 °C by sonication (5 seconds on and 5 seconds off) for 1 hour. The soluble fraction of the cell lysate was decanted after centrifugation at 10,000 × g for an hour at 4 °C. The insoluble inclusion bodies containing BM2 were resuspended in 15 mL lysis buffer containing 4 M urea and 0.2% (w/w) sodium dodecylsulfate (SDS) and shaken for 2 hr at 4 °C to solubilize the protein. The remaining inclusion bodies and cell debris were spun down by centrifugation for 1 hr at 10,000 × g at 4 °C. The supernatant was filtered through a 0.22 μm filter and loaded onto a gravity-flow chromatography column with ~5 mL nickel affinity resin (Profinity IMAC, BioRad). The solution was bound to the resin overnight by gentle shaking at 4 °C. 100 mL of wash buffer (pH 8.0, 50 mM Tris-HCl, 100 mM NaCl, 4 M urea and 0.1% (w/w) SDS) was used to wash the column, then BM2 was collected with 50 mL elution buffer (pH 8.0, 50 mM Tris-HCl, 50 mM NaCl 0.2 M imidazole, 0.05% (w/w) SDS). The crude protein concentration was determined by the 280 nm absorbance using a molar extinction coefficient of 6970 M^−1^ cm^−1^. The typical crude protein yield was 40–50 mg per liter of M9 media.

The eluted protein (~20 mL) was concentrated to ~5 mL using a 3 kDa Amicon filter at 4500 × g and 4 °C. To remove SDS and imidazole, we dialyzed BM2(1–51) against 1 L of Milli-Q water at room temperature using 3.5 kDa dialysis tubing (Spectrum Labs) for 4–5 days with 2 water changes per day. The protein precipitated after ~2 days, indicating loss of detergent over time. Subsequently, we used reversed-phase HLPC to further purify the protein, because the N-terminal tag could not be cleaved from BM2(1–51) using TEV protease. We attempted to solubilize the protein in a variety of detergents including DPC, OG, CHAPS, Triton X-100, DDM, LDAO, SDS and DHPC, and with various additives including urea, glycerol, and EDTA. However, we did not find a condition under which both the protein was soluble and the TEV cleavage was effective. Preparative reversed-phase HPLC was carried out on a Varian ProStar 210 System using a Vydac C18 column (10 μm particle size, 22 mm × 250 mm). The protein was dissolved in 30%:70% (v/v) acetonitrile: water containing 0.1% (v/v) trifluoroacetic acid (TFA) and eluted with a linear gradient of 35–99% acetonitrile over 70 minutes at a flow rate of 10 mL/min. The mass (9800.21 Da for unlabeled protein) and purity (>95%) of the protein was verified using MALDI mass spectrometry. The purified protein was lyophilized and stored at −30 °C.

### Preparation of proteoliposome samples

Two proteoliposome samples are used in this study. Most experiments used 1,2-dilauroyl-sn-glycero-3-phosphoethanolamine (DLPE) to reconstitute the protein. About 15 mg protein was dissolved in 1.5 mL trifluoroethanol (TFE) and mixed with 15 mg DLPE in 750 μL 90%: 10% (v/v) chloroform: methanol. The organic solvents were removed under a stream of nitrogen gas, and the film was further dried under vacuum at room temperature overnight. The film was resuspended in 4 mL of pH 7.5 buffer (20 mM Tris-HCl, 2 mM ethylenediaminetetraacetic acid (EDTA) and 0.2 mM NaN_3_) by vortexing and sonicating 2–3 times for 2 min each until a homogeneous suspension was obtained. The sample was then freeze-thawed 7 times between a 39 °C water bath and liquid nitrogen. The proteoliposome sample was centrifuged for 3 hr at 40,000 rpm and 4 °C to obtain a homogeneous membrane pellet. The pellet was allowed to dry overnight in a desiccator to a final hydration level of ~40% by mass and then packed into a 3.2 mm thin-wall rotor for solid-state NMR experiments. To investigate if the protein chemical shifts are sensitive to the membrane environment, we also prepared a second membrane sample, which consisted of 3.2 mg BM2(1–51) mixed with 4.8 mg of 4:1 (w/w) 1-palmitoyl-2-oleoyl-glycero-3-phosphocholine (POPC):1-palmitoyl-2-oleoyl-sn-glycero-3-phospho-(1′-rac-glycerol) (POPG). The sample was prepared in an identical manner, except that the volume of the organic solvent was reduced to keep the protein and lipid concentrations at 10 mg/mL and 20 mg/mL, respectively.

### Circular dichroism (CD) experiments

CD spectra were measured at room temperature on an AVIV 202 spectrophotometer using a 1 mm path-length quartz cuvette. Three scans were co-added for each spectrum. For the micelle sample, the protein was dissolved in an *n*-dodecylphosphocholine (DPC) buffer (pH 7.5, 10 mM NaHPO_4_, 0.5% w/w DPC) at a protein concentration of 0.15 mg/mL (15 μM). The protein-free buffer spectrum was subtracted from the protein-containing spectrum. For the bilayer sample, POPC:POPG vesicles were dissolved in 3 mL of CD buffer (pH 7.5, 10 mM NaHPO_4_, 1.5 mg/mL total lipids) and the protein concentration was 1.0 mg/mL. This proteoliposome was freeze-thawed 7 times and bath-sonicated for ~1 hour to increase optical transparency. The solution was diluted to 0.2 mg/mL (20 μM) protein before the measurement. A protein-free control sample was prepared identically, and its spectrum was subtracted from the spectrum of the protein-containing sample. The CD spectral intensities are reported as ellipticity in units of millidegrees. Deconvolution was conducted using the BestSel web server^69^. The spectral deconvolution result is highly sensitive to the protein concentration. To account for potential inaccuracies in the protein concentration determination, we allowed fitting of the spectral amplitude factor in BestSel. This yielded more accurate fits such that the final fitted CD spectra were indistinguishable from the experimental data. The final amplitude factors used were 1 for the DPC micelle data and 2 for the POPC:POPG vesicle data.

### Solid-state NMR experiments and data analysis

All solid-state NMR spectra of the BM2/DLPE sample were measured on Bruker Avance II 800 MHz (18.8 T) and 900 MHz (21.1 T) NMR spectrometers using 3.2 mm HCN probes. Magic-angle-spinning (MAS) frequencies were 14 kHz for the 800 MHz experiments and 15.75 kHz for the 900 MHz experiments. Typical radiofrequency (RF) field strengths were 50–71 kHz for ^1^H, 50 kHz for ^13^C, and 33–42 kHz for ^15^N. Reported sample temperatures are direct readings from the probe thermocouple, while the actual sample temperatures are estimated to be 10–15 K higher at the MAS frequencies used. One- and two-dimensional NMR spectra of the POPC: POPG sample were measured on a Bruker Avance III HD 600 MHz (14.1 T) spectrometer using a 1.9 mm MAS probe. The sample was spun at 12 kHz and the sample temperature was either 260 K or 290 K. The RF field strengths were 63–83 kHz for ^1^H, 63 kHz for ^13^C, and 42 kHz for ^15^N. Typical recycle delays for all experiments were 1.5–3.0 s.

^13^C chemical shifts were referenced externally to the adamantane CH_2_ chemical shift at 38.48 ppm on the tetramethylsilane scale and ^15^N chemical shifts were referenced to the ^15^N peak of N-acetylvaline at 122.00 ppm on the liquid ammonia scale. All spectra were processed using the Bruker TopSpin package and chemical shift assignment was conducted in Sparky^70^. (ϕ, ψ) torsion angles were calculated using the TALOS-N software package^71^ after ^13^C chemical shifts were converted to the DSS scale by adding 2.00 ppm^72^.

1D ^13^C and ^15^N cross polarization (CP), direct polarization (DP) and refocused Insensitive Nuclei Enhanced by Polarization Transfer (INEPT) spectra were measured using recycle delays of 1.5–2 s, 3–5 s, and 2 s, respectively. Acquisition times were 14–18 ms for CP, 25 ms for DP, and 30–37 ms for INEPT. The INEPT delays were chosen to be 1.6 ms and 1.1 ms to keep all ^13^C signals positive. 2D ^13^C-^13^C correlation experiments were conducted using COmbined $R{2}_{n}^{v}$-Driven (CORD) mixing for ^13^C-^13^C spin diffusion^73^. The 2D ^13^C-^13^C correlation spectrum of the POPC: POPG sample was measured using 52 ms CORD at 260 K under 12 kHz MAS on the 600 MHz spectrometer. 2D ^15^N-^13^C and 3D ^15^N-^13^C-^13^C correlation spectra for sequential resonance assignment were measured using the out-and-back Transferred-Echo Double Resonance (TEDOR) pulse sequence^74^ and the SPECtrally Induced Filtering In Combination with Cross Polarization (SPECIFIC-CP^75^) sequence for ^15^N-^13^C polarization transfer^76^. Detailed experimental conditions for the 2D and 3D spectra of the DLPE-bound BM2 sample are given in Supplementary Table S4.

Secondary shifts for amide protons were calculated using published random coil chemical shifts^58^, but very similar results were also observed using TALOS-N. The periodicity of H^N^ secondary chemical shifts in coiled coils were fit using $\partial {{\rm{H}}}_{\exp }^{N}-\partial {{\rm{H}}}_{rc}^{N}=O+A\,\sin (2\pi x/h+p)$, where *O* is the vertical offset, *A* is the amplitude, *x* is the residue number, *h* is the number of residues per turn, and *p* is the phase of the sinusoidal wave. For GCN4, bovine IF1 and myosin-binding subunit, all four parameters were fit and similar results of *O* = −0.09–0.07 ppm, *A* = 0.29–0.55 ppm, *h* = 3.45–3.53 residues/turn were obtained, while *p* matched the phase expected from the structures. For BM2, *h* and *p* did not fit to ~3.5 residues/turn and the expected phase, so we manually restricted them and only fit *A* and *O*. ^15^N secondary shifts were not periodic in GCN4 (data not shown).

## Supplementary information

Supporting Information

## References

[1] Murphy SL, Xu J, Kochanek KD, Curtin SC, Arias E (2017). Deaths: Final Data for 2015. Natl. Vital Stat. Rep..

[2] Pinto LH, Holsinger LJ, Lamb RA (1992). Influenza virus M2 protein has ion channel activity. Cell.

[3] Pinto LH, Lamb RA (2006). The M2 proton channels of influenza A and B viruses. J. Biol. Chem..

[4] Watanabe S, Imai M, Ohara Y, Odagiri T (2003). Influenza B Virus BM2 Protein Is Transported through the trans-Golgi Network as an Integral Membrane Protein. J. Virol..

[5] Hay AJ, Wolstenholme AJ, Skehel JJ, Smith MH (1985). The molecular basis of the specific anti-influenza action of amantadine. EMBO J..

[6] Wang C, Takeuchi K, Pinto LH, Lamb RA (1993). Ion channel activity of influenza A virus M2 protein: characterization of the amantadine block. J Virol..

[7] Stouffer AL (2008). Structural basis for the function and inhibition of an influenza virus proton channel. Nature.

[8] Cady SD (2010). Structure of the amantadine binding site of influenza M2 proton channels in lipid bilayers. Nature.

[9] Pinto LH (1997). A functionally defined model for the M2 proton channel of influenza A virus suggests a mechanism for its ion selectivity. Proc. Natl. Acad. Sci. USA.

[10] Ma C (2008). Identification of the pore-lining residues of the BM2 ion channel protein of influenza B virus. J. Biol. Chem..

[11] Mandala VS, Liao SY, Kwon B, Hong M (2017). Structural Basis for Asymmetric Conductance of the Influenza M2 Proton Channel Investigated by Solid-State NMR Spectroscopy. J. Mol. Biol..

[12] Betakova T, Hay AJ (2009). Comparison of the activities of BM2 protein and its H19 and W23 mutants of influenza B virus with activities of M2 protein and its H37 and W41 mutants of influenza A virus. Arch. Virol..

[13] Wang C, Lamb RA, Pinto LH (1995). Activation of the M2 ion channel of influenza virus: a role for the transmembrane domain histidine residue. Biophys. J..

[14] Balannik V (2010). Functional studies and modeling of pore-lining residue mutants of the influenza a virus M2 ion channel. Biochemistry.

[15] Wang J, Pielak RM, McClintock MA, Chou JJ (2009). Solution structure and functional analysis of the influenza B proton channel. Nat. Struct. Mol. Biol..

[16] Mould JA (2003). Influenza B virus BM2 protein has ion channel activity that conducts protons across membranes. Dev. Cell.

[17] Williams JK, Tietze D, Lee M, Wang J, Hong M (2016). Solid-State NMR Investigation of the Conformation, Proton Conduction, and Hydration of the Influenza B Virus M2 Transmembrane Proton Channel. J. Am. Chem. Soc..

[18] Williams JK, Shcherbakov AA, Wang J, Hong M (2017). Protonation equilibria and pore-opening structure of the dual-histidine influenza B virus M2 transmembrane proton channel from solid-state NMR. J. Biol. Chem..

[19] Zhou HX, Cross TA (2013). Influences of membrane mimetic environments on membrane protein structures. Annu. Rev. Biophys..

[20] Hong M, DeGrado WF (2012). Structural basis for proton conduction and inhibition by the influenza M2 protein. Protein Sci..

[21] Acharya A (2010). Structural mechanism of proton transport through the influenza A M2 protein. Proc. Natl. Acad. Sci. USA.

[22] Thomaston JL (2015). High-resolution structures of the M2 channel from influenza A virus reveal dynamic pathways for proton stabilization and transduction. Proc. Natl. Acad. Sci. USA.

[23] Thomaston JL (2017). XFEL structures of the influenza M2 proton channel: Room temperature water networks and insights into proton conduction. Proc. Natl. Acad. Sci. USA.

[24] Sharma M (2010). Insight into the mechanism of the influenza A proton channel from a structure in a lipid bilayer. Science.

[25] Schnell JR, Chou JJ (2008). Structure and mechanism of the M2 proton channel of influenza A virus. Nature.

[26] Andreas LB (2015). Structure and Mechanism of the Influenza A M218-60 Dimer of Dimers. J. Am. Chem. Soc..

[27] Jaakola VP (2008). The 2.6 Angstrom Crystal Structure of a Human A(2A) Adenosine Receptor Bound to an Antagonist. Science.

[28] Doyle DA (1998). The structure of the potassium channel: Molecular basis of K+ conduction and selectivity. Science.

[29] Jiang YX (2002). The open pore conformation of potassium channels. Nature.

[30] Shcherbakov AA, Hong M (2018). Rapid Measurement of Long-Range Distances in Proteins by Multidimensional ^13^C-19F REDOR NMR under Fast Magic-Angle Spinning. J. Biomol. NMR..

[31] Chipot C (2018). Perturbations of Native Membrane Protein Structure in Alkyl Phosphocholine Detergents: A Critical Assessment of NMR and Biophysical Studies. Chem. Rev..

[32] Balannik V, Lamb RA, Pinto LH (2008). The oligomeric state of the active BM2 ion channel protein of influenza B virus. J. Biol. Chem..

[33] Paterson RG, Takeda M, Ohigashi Y, Pinto LH, Lamb RA (2003). Influenza B virus BM2 protein is an oligomeric integral membrane protein expressed at the cell surface. Virology.

[34] Greenfield NJ (2006). Using circular dichroism spectra to estimate protein secondary structure. Nat. Protoc..

[35] Baumruck AC, Tietze D, Steinacker LK, Tietze AA (2018). Chemical synthesis of membrane proteins: a model study on the influenza virus B proton channel. Chem. Sci..

[36] Lee M, Hong M (2014). Cryoprotection of lipid membranes for high-resolution solid-state NMR studies of membrane peptides and proteins at low temperature. J. Biomol. NMR.

[37] Liao SY, Lee M, Wang T, Sergeyev IV, Hong M (2016). Efficient DNP NMR of membrane proteins: sample preparation protocols, sensitivity, and radical location. J. Biomol. NMR.

[38] Hu F, Luo W, Cady SD, Hong M (2011). Conformational plasticity of the influenza A M2 transmembrane helix in lipid bilayers under varying pH, drug binding, and membrane thickness. Biochim. Biophys. Acta.

[39] Mandala VS, Gelenter MD, Hong M (2018). Transport-Relevant Protein Conformational Dynamics and Water Dynamics on Multiple Time Scales in an Archetypal Proton Channel: Insights from Solid-State NMR. J. Am. Chem. Soc..

[40] Liao SY, Yang Y, Tietze D, Hong M (2015). The influenza m2 cytoplasmic tail changes the proton-exchange equilibria and the backbone conformation of the transmembrane histidine residue to facilitate proton conduction. J. Am. Chem. Soc..

[41] Cady SD, Hong M (2008). Amantadine-Induced Conformational and Dynamical Changes of the Influenza M2 Transmembrane Proton Channel. Proc. Natl. Acad. Sci. USA.

[42] Spera S, Bax A (1991). Empirical Correlation between Protein Backbone Conformation and C-Alpha and C-Beta C-13 Nuclear-Magnetic-Resonance Chemical-Shifts. J. Am. Chem. Soc..

[43] Wishart DS, Sykes BD, Richards FM (1991). Relationship between Nuclear-Magnetic-Resonance Chemical-Shift and Protein Secondary Structure. J. Mol. Biol..

[44] Nagle JF, Tristram-Nagle S (2000). Structure of lipid bilayers. Biochim. Biophys. Acta.

[45] Kucerka N, Nieh MP, Katsaras J (2011). Fluid phase lipid areas and bilayer thicknesses of commonly used phosphatidylcholines as a function of temperature. Biochim. Biophys. Acta.

[46] Cady SD, Goodman C, Tatko C, DeGrado WF, Hong M (2007). Determining the orientation of uniaxially rotating membrane proteins using unoriented samples: a ^2^H, ^13^C, and ^15^N solid-state NMR investigation of the dynamics and orientation of a transmembrane helical bundle. J. Am. Chem. Soc..

[47] Duong-Ly KC, Nanda V, DeGrado WF, Howard KP (2005). The conformation of the pore region of the M2 proton channel depends on lipid bilayer environment. Protein Sci..

[48] De Planque MR (2002). The effects of hydrophobic mismatch between phosphatidylcholine bilayers and transmembrane alpha-helical peptides depend on the nature of interfacially exposed aromatic and charged residues. Biochemistry.

[49] Harbury PB, Kim PS, Alber T (1994). Crystal structure of an isoleucine-zipper trimer. Nature.

[50] Blundell T, Barlow D, Borkakoti N, Thornton J (1983). Solvent-induced distortions and the curvature of alpha-helices. Nature.

[51] O’Shea EK, Klemm JD, Kim PS, Alber T (1991). X-ray structure of the GCN4 leucine zipper, a two-stranded, parallel coiled coil. Science.

[52] Chou JJ, Kaufman JD, Stahl SJ, Wingfield PT, Bax A (2002). Micelle-induced curvature in a water-insoluble HIV-1 Env peptide revealed by NMR dipolar coupling measurement in stretched polyacrylamide gel. J. Am. Chem. Soc..

[53] Koirala S (2010). Molecular architecture of a dynamin adaptor: implications for assembly of mitochondrial fission complexes. J. Cell Biol..

[54] Bhate MP (2018). Structure and Function of the Transmembrane Domain of NsaS, an Antibiotic Sensing Histidine Kinase in Staphylococcus aureus. J. Am. Chem. Soc..

[55] Goodman EM, Kim PS (1991). Periodicity of amide proton exchange rates in a coiled-coil leucine zipper peptide. Biochemistry.

[56] Walsh ST (2003). The hydration of amides in helices; a comprehensive picture from molecular dynamics, IR, and NMR. Prot. Sci..

[57] Nikolaev Y, Pervushin K (2007). NMR spin state exchange spectroscopy reveals equilibrium of two distinct conformations of leucine zipper GCN4 in solution. J. Am. Chem. Soc..

[58] Wang YJ, Jardetzky O (2002). Probability-based protein secondary structure identification using combined NMR chemical-shift data. Prot. Sci..

[59] Wagner G, Pardi A (1983). & Wuthrich, K. Hydrogen-Bond Length and H-1-Nmr Chemical-Shifts in Proteins. J. Am. Chem. Soc..

[60] Gordon-Smith DJ (2001). Solution structure of a C-terminal coiled-coil domain from bovine IF(1): the inhibitor protein of F(1) ATPase. J. Mol. Biol..

[61] Sharma AK, Birrane G, Anklin C, Rigby AC, Alper SL (2017). NMR insight into myosin-binding subunit coiled-coil structure reveals binding interface with protein kinase G-Ialpha leucine zipper in vascular function. J. Biol. Chem..

[62] Lipfert J, Columbus L, Chu VB, Lesley SA, Doniach S (2007). Size and shape of detergent micelles determined by small-angle X-ray scattering. J. Phys. Chem. B.

[63] Tausk RJ, van Esch J, Karmiggelt J, Voordouw G, Overbeek JT (1974). Physical chemical studies of short-chain lecithin homologues. II. Micellar weights of dihexanoyl- and diheptanoyllecithin. Biophys. Chem..

[64] Van Horn WD (2009). Solution Nuclear Magnetic Resonance Structure of Membrane-Integral Diacylglycerol Kinase. Science.

[65] Chen YK (2014). Conformation and Topology of Diacylglycerol Kinase in E. coli Membranes Revealed by Solid-state NMR Spectroscopy. Angew. Chemie. Int. Ed. Engl..

[66] Li DF (2013). Crystal structure of the integral membrane diacylglycerol kinase. Nature.

[67] Koehler J (2010). Lysophospholipid Micelles Sustain the Stability and Catalytic Activity of Diacylglycerol Kinase in the Absence of Lipids. Biochemistry.

[68] Page RC, Lee S, Moore JD, Opella SJ, Cross TA (2009). Backbone structure of a small helical integral membrane protein: A unique structural characterization. Prot. Sci..

[69] Micsonai A (2015). Accurate secondary structure prediction and fold recognition for circular dichroism spectroscopy. Proc. Natl. Acad. Sci. USA.

[70] Goddard, T. D. & Kneller, D. G. SPARKY 3. *University of California*, *San Francisco,* (2007).

[71] Shen Y, Bax A (2013). Protein backbone and sidechain torsion angles predicted from NMR chemical shifts using artificial neural networks. J. Biomol. NMR.

[72] Harris RK (2008). Further conventions for NMR shielding and chemical shifts (IUPAC recommendations 2008). Pure Appl. Chem..

[73] Hou GJ, Yan S, Trebosc J, Amoureux JP, Polenova T (2013). Broadband homonuclear correlation spectroscopy driven by combined R2(n)(v) sequences under fast magic angle spinning for NMR structural analysis of organic and biological solids. J. Magn. Reson..

[74] Daviso E, Eddy MT, Andreas LB, Griffin RG, Herzfeld J (2013). Efficient resonance assignment of proteins in MAS NMR by simultaneous intra- and inter-residue 3D correlation spectroscopy. J. Biomol. NMR.

[75] Baldus M, Petkova AT, Herzfeld J, Griffin RG (1998). Cross polarization in the tilted frame: assignment and spectral simplification in heteronuclear spin systems. Mol. Phys..

[76] Hung I, Gan ZH (2015). Spin-locking and cross-polarization under magic-angle spinning of uniformly labeled solids. J. Magn. Resn..

