# Supplementary material for: The Transmembrane Conformation of the Influenza B Virus M2 Protein in Lipid Bilayers
Source: Sci Rep. 2019 Mar 6;9:3725. doi: 10.1038/s41598-019-40217-1 (PMC6403292; doi:10.1038/s41598-019-40217-1)
Supplement: Supplementary file 1 — Supporting Information [file 41598_2019_40217_MOESM1_ESM.pdf]

Supplementary Information

The Transmembrane Conformation of the Influenza B Virus M2 Protein in Lipid Bilayers

Venkata S. Mandala, Shu-Yu Liao, Martin D. Gelenter, and Mei Hong *

Department of Chemistry, Massachusetts Institute of Technology, 170 Albany Street, Cambridge, MA 02139

* Corresponding author, email: meihong@mit.edu

Table S1. ^{13}C and ^{15}N chemical shifts (ppm) of BM2(1-51) bound to DLPE bilayers. ^{15}N chemical shifts are referenced to liquid ammonia and ^{13}C chemical shifts are referenced to TMS. Ile residues are not labeled and therefore their chemical shifts are unassigned.

Residue	Cα	Cβ	C'	N	Cγ/γ1	Cγ2	Cδ/δ1	Cδ2/ε/ε2	Cζ	Nδ2/Nε/ Nε2	Nζ/Nη1
Met1	52.3	35.2	171.7	123.9	30.2		19.3				
Phe2	56.4	37.1									
Glu3	54.7	28.1			34.2		181.6				
Pro4	60.9	30.6			25.1		48.2				
Phe5	55.9	35.3	173.0	117.5			129.5		128.3		
Gln6	56.1	24.0	176.1	116.2	29.6						
Ile7											
Leu8	56.2	39.6	175.7	118.6	24.4		23.9	21.2			
Ser9	60.1	60.9	174.3	116.0							
Ile10											
Cys11	57.3	24.8	176.6	118.3							
Ser12	60.3	60.3	175.9	115.3							
Phe13	59.1	37.2	175.5	120.4			128.9		128.9		
Ile14											
Leu15	56.3	39.2	176.7	120.1	24.9		23.8	22.7			
Ser16	60.0	60.7	174.5	115.6							
Ala17	53.3	15.8	177.4	125.2							
Leu18	56.3	38.4	176.7	119.0	24.7		24.2	21.4			
His19	57.1	27.0		120.0				116.9			
Phe20	59.3	36.6	175.9	121.6			129.3		129.3		
Met21	57.3	32.6	176.1	122.1	30.8		19.5				
Ala22	53.6	16.4	177.6	122.3							
Trp23	59.7	27.8	177.3	120.5	109.8						
Thr24	65.6	66.1	174.3	118.7		19.6					
Ile25											
Gly26	45.2		173.9	107.0							
His27	54.5	25.6	174.2	117.0				117.9			
Leu28	56.4	39.1									
Asn29	51.3	37.0									
Gln30											
Ile31											
Lys32	52.91	32.93	172.2	126.2	23.1		27.3	40.3			32.8
Arg33	52.55	31.28	173.3	128.3	25.5		41.7	157.5		72.2	85.0
Val35	58.65	32.55			19.3		19.3				
Pro44	64.48	31.63			26.1		48.3				

Table S2. Comparison of ^{15}N chemical shifts (ppm) of BM2(1-51) bound to DLPE bilayers and of BM2(1-33) in DHPC micelles. Only residues where ^{15}N chemical shifts are available for comparison in both studies are given. The construct in DHPC micelles included two mutations to the native sequence, C11S and M21I, thus these residues are excluded from the comparison. ^{15}N chemical shifts are referenced on the liquid ammonia scale.

**Residue**	δN_{DLPE} **(ppm)**	δN_{DHPC} **(ppm)**	$\delta N_{DLPE} - \delta N_{DHPC}$ **(ppm)**
Phe5	117.5	116.1	1.4
Gln6	116.2	120.8	-4.6
Leu8	118.6	122.5	-3.9
Ser9	116.0	117.0	-1.0
Ser12	115.3	117.4	-2.1
Phe13	120.4	123.7	-3.3
Leu15	120.1	119.2	0.9
Ser16	115.6	116.1	-0.5
Ala17	125.2	124.9	0.3
Leu18	119.0	117.7	1.3
His19	120.0	119.3	0.7
Phe20	121.6	120.3	1.3
Ala22	122.3	122.6	-0.3
Trp23	120.5	120.3	0.2
Thr24	118.7	117.1	1.6
Gly26	107.0	107.1	-0.1
His27	117.0	120.0	-3.0
Lys32	126.2	124.2	2.0

Table S3. Backbone (φ, ψ) torsion angles predicted from measured ^{13}C and ^{15}N chemical shifts using TALOS-N. Error bars represent the precision of the TALOS-N prediction.

Residue	φ Angle	ψ Angle
Pro4	-61.4 ± 6.5°	146.3 ± 5.0°
Phe5	-64.6 ± 12.3°	-33.8 ± 11.2°
Gln6	-58.4 ± 24.1°	-32.0 ± 16.3°
Ile7	-70.0 ± 4.6°	-36.8 ± 5.9°
Leu8	-66.9 ± 4.7°	-36.3 ± 5.7°
Ser9	-66.6 ± 4.9°	-38.0 ± 5.5°
Ile10	-66.2 ± 5.8°	-39.0 ± 6.3°
Cys11	-64.6 ± 3.9°	-35.3 ± 6.3°
Ser12	-65.8 ± 4.9°	-36.7 ± 4.6°
Phe13	-66.1 ± 5.1°	-38.8 ± 6.5°
Ile14	-68.9 ± 4.2°	-36.7 ± 5.3°
Leu15	-66.0 ± 5.5°	-35.7 ± 5.1°
Ser16	-66.7 ± 4.0°	-37.5 ± 6.6°
Ala17	-65.0 ± 6.6°	-39.4 ± 5.0°
Leu18	-67.6 ± 4.5°	-38.6 ± 5.0°
His19	-66.4 ± 3.6°	-38.5 ± 3.9°
Phe20	-67.2 ± 5.4°	-37.2 ± 5.3°
Met21	-65.2 ± 4.3°	-40.7 ± 4.5°
Ala22	-65.6 ± 5.0°	-35.4 ± 6.3°
Trp23	-62.9 ± 4.6°	-44.1 ± 5.4°
Thr24	-66.8 ± 4.2°	-38.7 ± 4.5°
Ile25	-67.1 ± 4.9°	-34.5 ± 4.9°
Gly26	-65.7 ± 4.0°	-35.2 ± 7.2°
His27	-70.7 ± 10.0°	-33.1 ± 13.8°
Lys32	-98.9 ± 15.0°	134.2 ± 9.5°
Arg33	-116.2 ± 14.0°	137.2 ± 12.4°

Table S4. Experimental conditions for the 2D and 3D ^{13}C-^{13}C and ^{15}N-^{13}C correlation spectra for DLPE-bound BM2(1-51). Typical ^{1}H decoupling rf field strengths were 71 kHz. The 2D NCA and 2D N(CO)CX were measured at the National High Magnetic Field Laboratory.

Experiment	^{1}H Larmor frequency, MAS rate, Temperature	Mixing scheme and time, rf field strengths	Number of scans, Recycle delays, Digitization	Expt. time
2D CC	800 MHz, 14 kHz, 285 K	55 ms CORD	NS = 64, D1 = 1.8 s, TD1 = 500, SW1 = 198.8 ppm, IN1 = 25 μs	17 hrs
2D CC	900 MHz, 15.75 kHz, 270 K	100 ms CORD	NS = 192, D1 = 1.5 s, TD1 = 200, SW1 = 200 ppm, IN1 = 22.1 μs	18 hrs
2D CC	800 MHz, 14 kHz, 285 K	300 ms CORD	NS = 128, D1 = 2.2 s, TD1 = 450, SW1 = 198.8 ppm, IN1 = 25 μs	40 hrs
2D NCA	800 MHz, 14 kHz, 260 K	4 ms ^{SPECIFIC}CP, ^{15}N @ 35 kHz, ^{13}C @ 21 kHz, ^{1}H @ 100 kHz	NS = 256, D1 = 1.5 s, TD1 = 60, SW1 = 49.3 ppm, IN1 = 250 μs	7 hrs
2D N(CA)CX	800 MHz, 14 kHz, 285 K	1.43 ms TEDOR, ^{15}N @ 33 kHz, ^{13}C @ 50 kHz, ^{1}H @ 71 kHz, 110 ms CORD	NS = 288, D1 = 2.0 s, TD1 = 220, SW1 = 172.7 ppm, IN1 = 71.4 μs	38 hrs
2D N(CO)CX	800 MHz, 14 kHz, 260 K	4 ms ^{SPECIFIC}CP, ^{15}N @ 35 kHz, ^{13}C @ 49 kHz, ^{1}H @ 100 kHz, 150 ms DARR	NS = 760, D1 = 1.5 s, TD1 = 40, SW1 = 49.3 ppm, IN1 = 250 μs	14 hrs
3D NCACX	900 MHz, 15.75 kHz, 270 K	4 ms ^{SPECIFIC}CP, ^{15}N @ 28 kHz, ^{13}C @ 12 kHz, ^{1}H @ 90 kHz, 110 ms CORD	NS = 224, D1 = 1.8 s, TD1 = 32, SW1 = 35.1 ppm, IN1 = 126 μs, TD2 = 24, SW2 = 40.3 ppm, IN2 = 272 μs	93 hrs
3D NCOCX	900 MHz, 15.75 kHz, 270 K	4 ms ^{SPECIFIC}CP, ^{15}N @ 24 kHz, ^{13}C @ 40 kHz, ^{1}H @ 90 kHz, 110 ms CORD	NS = 352, D1 = 1.9 s, TD1 = 18, SW1 = 20.1 ppm, IN1 = 220 μs, TD2 = 24, SW2 = 40.3 ppm, IN2 = 272 μs	87 hrs

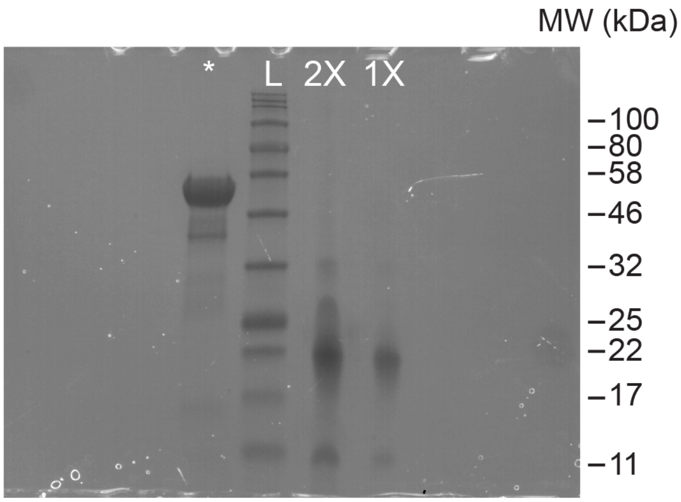

Figure S1. Full view of SDS-PAGE gel in main text Figure 2. Lanes marked with 2X and 1X are the BM2-containing lanes. The lane marked L is the ladder, and the lane marked with an asterisk (*) is a different protein.

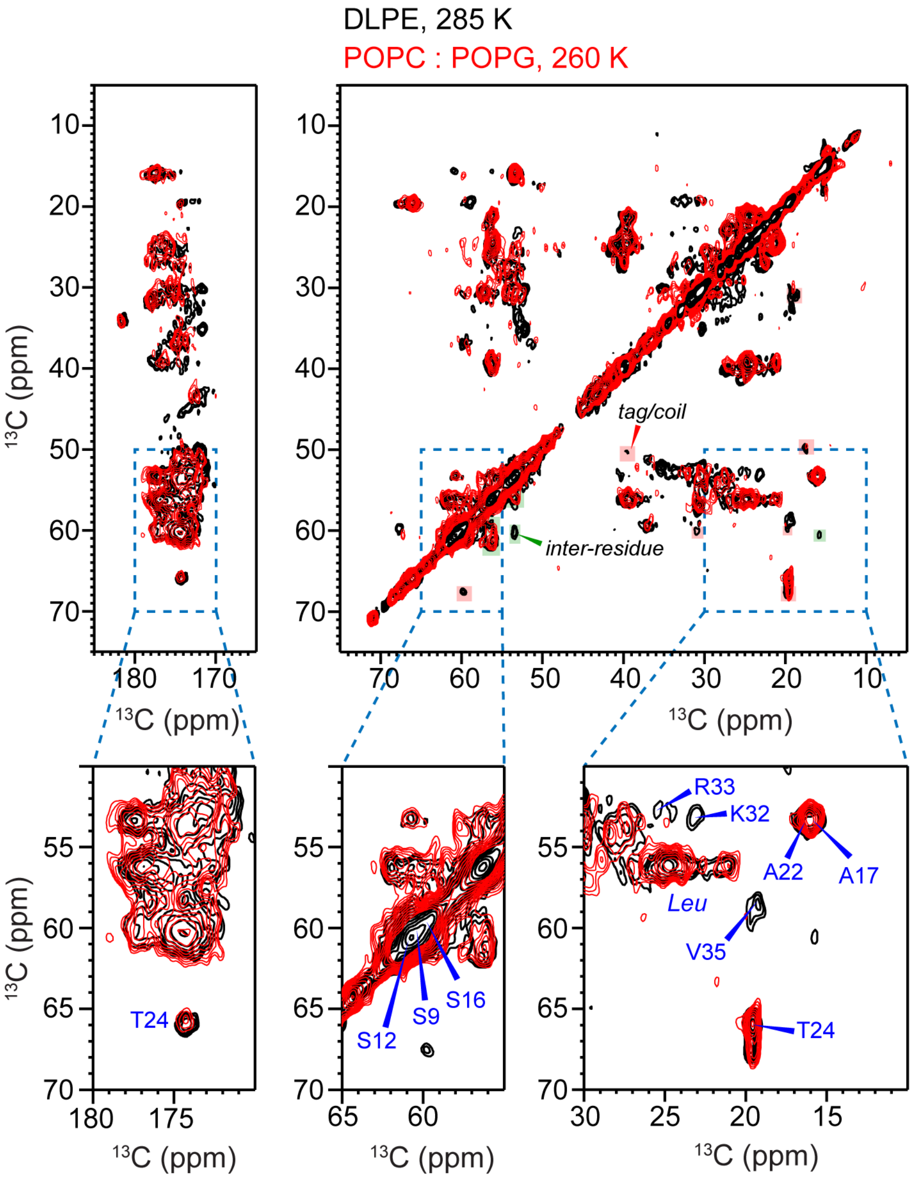

Figure S2. 2D ^{13}C-^{13}C correlation spectra of BM2 in DLPE (*black*) and 4:1 POPC : POPG (*red*) bilayers, indicating that the conformation of core TM residues is the same in the two membranes. The spectrum of the DLPE sample was measured using 55 ms CORD mixing, 14 kHz MAS at 285 K on the 800 MHz spectrometer in a 3.2 mm rotor. The spectrum of the POPC : POPG sample was measured using 52 ms CORD mixing, 12 kHz MAS at 260 K on a 600 MHz spectrometer in a 1.9 mm rotor. The DLPE sample shows more peaks, likely because the DLPC membrane is more rigid than the POPC : POPG membrane at the experimental temperature. But the chemical shifts of the TM residues are the same, as shown in the enlarged areas in the insets.
